# Gains in health insurance coverage explain variation in Democratic vote share in the 2008-2016 presidential elections

**DOI:** 10.1371/journal.pone.0214206

**Published:** 2019-04-04

**Authors:** Alex Hollingsworth, Aparna Soni, Aaron E. Carroll, John Cawley, Kosali Simon

**Affiliations:** 1 O’Neill School of Public and Environmental Affairs, Indiana University, Bloomington, IN, United States of America; 2 Kelley School of Business, Indiana University, Bloomington, IN, United States of America; 3 School of Medicine, Indiana University, Indianapolis, IN, United States of America; 4 Department of Policy Analysis and Management, Cornell University, Ithaca, NY, United States of America; 5 Department of Economics, Cornell University, Ithaca, NY, United States of America; 6 National Bureau of Economic Research, Cambridge, MA, United States of America; Augusta University, UNITED STATES

## Abstract

In the last decade, health care reform has dominated U.S. public policy and political discourse. Double-digit rate increases in premiums in the Health Insurance Marketplaces established by the Affordable Care Act (ACA) in 2018 make this an ongoing issue that could affect future elections. A seminal event that changed the course of policy and politics around health care reform is the 2016 presidential election. The results of the 2016 presidential election departed considerably from polling forecasts. Given the prominence of the Affordable Care Act in the election, we test whether changes in health insurance coverage at the county-level correlate with changes in party vote share in the presidential elections from 2008 through 2016. We find that a one-percentage-point increase in county health insurance coverage was associated with a 0.25-percentage-point increase in the vote share for the Democratic presidential candidate. We further find that these gains on the part of the Democratic candidate came almost fully at the expense of the Republican (as opposed to third-party) presidential candidates. We also estimate models separately for states that did and did not expand Medicaid and find no differential effect of insurance gains on Democratic vote share for states that expanded Medicaid compared to those that did not. Our results are consistent with the hypothesis that outcomes in health insurance markets played a role in the outcome of the 2016 presidential election. The decisions made by the current administration, and how those decisions affect health insurance coverage and costs, may be important factors in future elections as well.

## Introduction

Since 2010, close to 20 million Americans have gained health insurance [1]; however, the future of the Affordable Care Act (ACA) remains a dominant point of contention in US politics. Although efforts to repeal the ACA in part or as a whole have been ongoing since its passage, the level of activity increased following the 2016 presidential election, the results of which departed considerably from polling forecasts. The outcome of future presidential and midterm elections also may heavily depend on healthcare reform politics [2].

Although many issues and events influence national election outcomes, and one must be careful of overly simplistic explanations, one important factor discussed in the media is gains in health insurance coverage. The Affordable Care Act (ACA) was a major issue in the election [3], and changes in the health insurance coverage landscape leading up to the election may explain some of the geographic variation in the presidential vote, just as it may for future elections [2].

This hypothesis—that changes in health care coverage influenced the election—is testable. One source of information is exit polls. According to one such poll by POLITICO/Morning Consult, health care was one of the top three issues for voters, and 58% of respondents said that the ACA should be repealed [4]. However, exit polls often rely on small, idiosyncratic samples and may be biased by selective response.

Our approach is to use county-level data on voting and health insurance to test this hypothesis. Specifically, we test whether changes in health insurance coverage at the county-level correlate with changes in party vote share in presidential elections from 2008 through 2016. Analyzing these relationships may help us better understand the results of the 2016 election, how these factors may influence voting in the future, and the importance of health insurance coverage to voters.

## Data and methods

Using data at the county-year level, we regress the vote share of the Democratic presidential candidate on the insurance rate for the population aged 18–64 and below 400% of the federal poverty level (FPL). We focus on the working-age population because there is less variation in health insurance coverage among those under age 18 (who are almost all covered, either by Medicaid or private insurance) and those over age 65 (who are almost all covered by Medicare).

Our county-year data cover the last three presidential elections: 2008, 2012, and 2016. The models control for county and year fixed-effects, so the coefficient on health insurance coverage can be interpreted as the percentage point change in the vote share received by the Democratic presidential candidate associated with a one percentage point change in the health insurance rate in that county.

Although the model controls for county fixed-effects, which address all time-invariant unobserved variables that may differ across counties, we also control for important county characteristics that may change over time: unemployment rate, median income, percent of population in poverty, percent of population that is non-white, population density, and campaign spending. We weight observations by county population to ensure that counties with a larger number of residents receive greater weight in the regression and thus the results are more easily generalizable to the entire U.S. Because counties in the same state may have correlated errors, we cluster-correct the standard errors at the state-level; this is more conservative than clustering errors at the county-level because there are far fewer states than counties). One such reason that counties may have correlated errors is due to within-county correlated exposure to negative political advertisements. Previous work has documented that negative perceptions of the Affordable Care Act (which resulted in insurance expansions) are related to a distaste for government involvement in health care [5] and that political advertisements leading up to presidential elections are the primary reported source of information for most voters have regarding government involvement in health care and the ACA [6]. Since counties are differentially competitive in presidential elections, they will be subjected to differential amounts and types of political advertisements; therefore, it is important control for both county fixed-effects and the potential for correlation of the county’s error across time.

We examine several other dependent variables in addition to the change in the share of votes received by the Democratic presidential candidate. Because changes in the Democratic vote share could come from either the Republican vote share or third-party vote shares, we estimate models for those dependent variables as well. Finally, we estimate models of voter turnout; i.e. the number of votes cast in the county divided by the county population, because if all parties were motivated to vote by changes in health insurance markets, that might be detectable only in turnout, rather than in shares of the vote received by each party.

**Fig 1:**
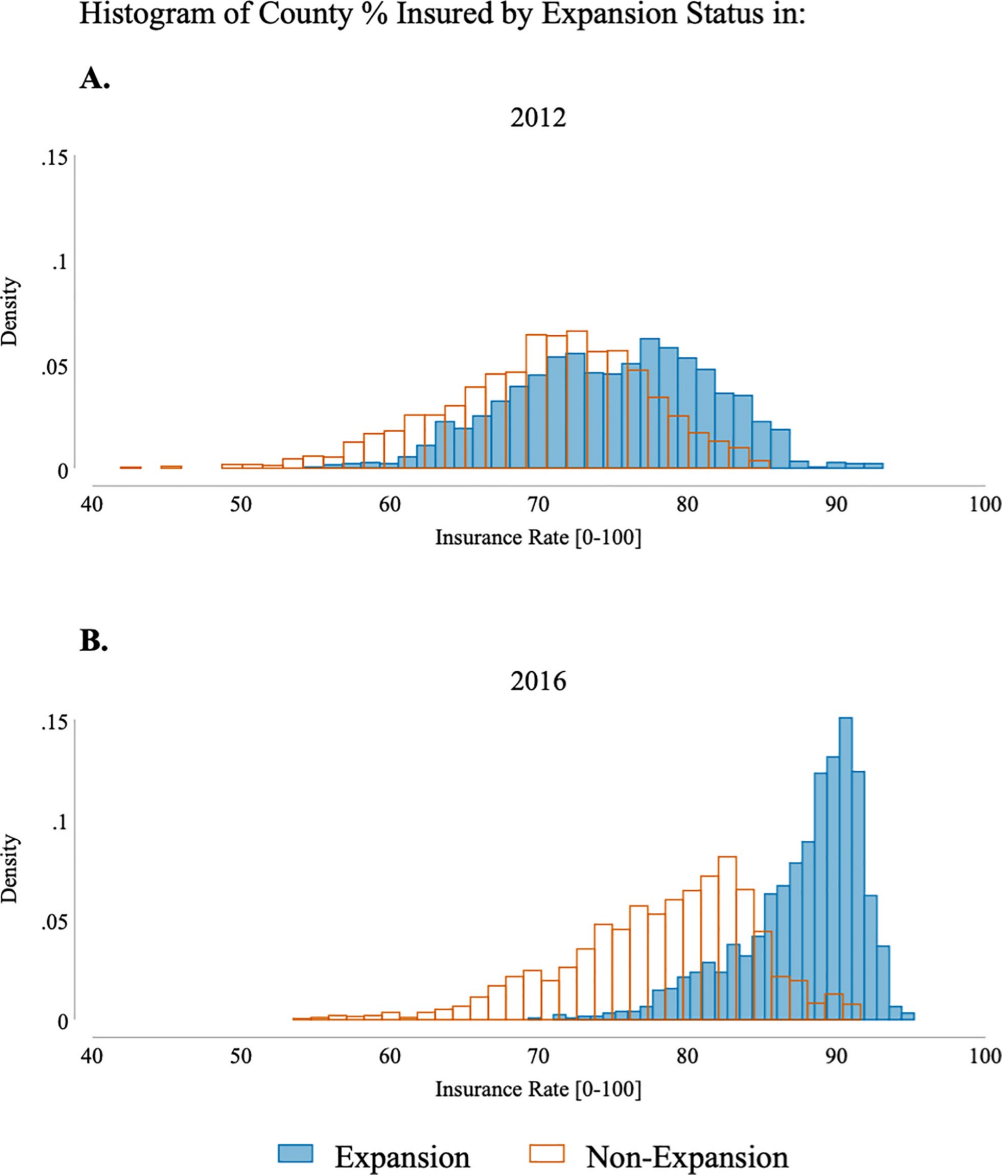
Distribution of health insurance coverage across time and medicaid expansion status. Insurance rate refers to the percent of the county's working-age population (18–64), below 400% FPL with health insurance. Medicaid expansion status is as reported by the Kaiser Family Foundation in Nov 2016.

Our main models are estimated for the entire U.S. However, we also estimate models separately by whether a state expanded its Medicaid program to low-income childless adults under the ACA. We do this for two reasons. First, Medicaid expansions are an important source of variation over time in health insurance coverage. Fig 1 shows the distribution of county health insurance rates for those below 400% of the federal poverty line in 2012 and in 2016. In each year a separate distribution is plotted for Medicaid expansion states and non-expansion states. Expansion is determined by 2016 status. Although there is substantial overlap, health insurance coverage tends to be higher in states that expanded Medicaid and health insurance coverage grew more in expansion states from 2012 to 2016 than in non-expansion states. Comparing the difference in distributions between expansion and non-expansion states from 2012 to 2016 highlights these facts. By estimating models separately for states that did and did not expand Medicaid we can test whether other sources of variation in health insurance are associated with party vote share in both types of states. Second, the Medicaid expansions themselves were politically controversial, so the relationship between the change in health insurance coverage and vote shares may differ in the expansion and non-expansion states.

Another potentially important issue affecting public sentiment is the cost of health insurance. ACA marketplace premiums rose a significant amount in many areas of the country in the past year, creating what some referred to as “rate shock” [7]. People who witnessed their insurance costs rise dramatically, and those who were inundated with national and local news stories about those spikes, might have been more likely to vote for the Republican candidate in 2016, especially since he campaigned vociferously that those rate increases indicated problems with Obamacare and campaigned on its repeal and replacement. We cannot estimate the effect of rising insurance premiums on election results using the models described above because the ACA marketplaces were established in 2014, so premiums data exists only for one presidential election year (2016). Nevertheless, in S1 Table, we provide suggestive evidence on the relationship between insurance premiums and election results by estimating models in which the *change* between 2012 and 2016 in the Republican share of the presidential vote is regressed on the recent *change* in ACA marketplace premiums.

Our hypothesis that increases in health insurance will influence county-level vote share for the Democratic Party is based upon the assumption that the constituents of each county behave in a manner that is accurately reflected by its county-level vote share. Our hypothesis is also based upon the idea that each constituent’s decision to vote, as well as the outcome of the vote, can be influenced by two channels: an indirect channel that captures the role of regional/national discussions and perceptions of the policy, and a direct channel that captures first-hand or near first-hand (e.g., community-level) experiences with the policy. The direct and indirect effects need not influence a policy in the same direction. If they influence voter behavior in contradictory directions, then it is not, a priori, clear which effect will dominate. To account for this potential confounding, we will control for both state and year fixed-effects. These should control for the role of the indirect channel and allow us, when examining the role of county-level impacts of insurance increases, to test for the presence and sign of the direct effects. We hypothesize that in national elections, after the indirect channel is accounted for, the communities that have benefited more from insurance expansions will have a higher vote share for the presidential candidate from the political party most likely to maintain or further increase insurance coverage: the Democratic Party. That is, we hypothesize that once regional and national confounders are accounted for, we will find a positive feedback between insurance expansions and the vote share for the Democratic Party at the county-level in U.S. presidential elections.

While there has been little work explicitly linking county-level voter decisions in elections to changes in insurance status, there is a substantial theoretical basis related to positive feedback on which our hypotheses are grounded. Pierson (1993) argued that new policies can create new politics and that there is the potential for such a positive feedback [8]. Pierson argued that these effects could come from either resource or interpretive effects. Resource effects are those that see policies as bundles of resources, which can impact the decisions of the mass public, states, and interest groups. Interpretive effects are those where the policy impacts politics by offering new information or a new way of thinking about an idea. Either of these channels is a plausible mechanism through which voters may decide to vote Democratic in response to increased local insurance coverage. We will not be able to separately identify either mechanism in this paper. However, we believe that it is likely that the resource effects will dominate as the benefits of insurance will be most salient to those who received expanded insurance due to the ACA, Medicaid expansion, or other insurance programs typically supported by Democratic candidates. In addition to this theoretical justification, recent empirical work by both Clinton and Sances (2018) and Haselswerdt (2017) finding that voter participation increased during 2014 congressional elections due to the Affordable Care Act further supports the likelihood of finding positive feedback between insurance expansions and Democratic vote share [9–10]. It should be mentioned that there is not a clear consensus that the insurance expansions should be expected to induce positive feedback effects. Patashnik and Zelizer (2013) argue that the effects of the ACA may not be sufficiently visible to create a positive feedback effect [11]. They state that the program complexity may have created a Mettler (2011) style submerged state in which program beneficiaries may not realize which political groups and/or parties are the progenitors of their insurance status [12]. If this is the case, it is possible that we will find no effect of local insurance expansions on voter behavior.

Data on election results by county are from the Guardian newspaper and Townhall (a political website), each of which make county-level U.S. election data available [13–14]. The source for county-level health insurance rates is the Small Area Health Insurance Estimates (SAHIE), county-level income and poverty rates are from the Small Area Income and Poverty Estimates (SAIPE), county unemployment rates are from the Bureau of Labor Statistics, and county-level populations by race and ethnicity are from the National Cancer Institute’s Surveillance, Epidemiology, and End Results Program (SEER).

We choose to use county-level data because the county is the smallest available geographic unit for which we could obtain actual voter data for U.S. presidential elections. However, because we use county-level data rather than individual-level data, it is possible that any analysis using such data could suffer from the ecological fallacy. That is, it is possible that findings from the county-level analyses would yield different results than a similar analysis that employed individual-level voting data linked to insurance status across time. We believe this to be an unlikely case since the county is a small geographic unit; it is the case, however, that any inferences made at the county-level from our findings are assumed to hold true at the individual-level. The problem would be exacerbated if we used larger geographic units such as states or regions. It is not possible to perform our analysis using the universe of election data from each presidential election since the United States neither records individual-level voting data nor links insurance status to voter specific information. Thus any individual-level analysis would be reliant on survey results, which would be from a small sample of the overall population. Any such survey data would not contain the full set of voter decisions and could contain problematic and difficult to detect biases. While it is a limitation of our paper that we do not have individual-level data, we believe it would be a greater limitation to forgo using the actual election data and restrict our data to a small, potentially biased, selection of survey results.

## Descriptive information

Table 1 provides population-weighted means of the variables in the regression model. In the 2016 election, the (population-weighted) Democratic vote share was 48.9 percent, down 2.2 percentage points from 2012. The Republican vote share was 46.1 percent, down 1.2 percentage points from 2012. Meanwhile, the vote share for other political parties increased by 3.4 percentage points from 1.6 percent in 2012 to 5.0 percent in 2016. Although the insurance rate decreased slightly from 2008 to 2012, it increased significantly from 70.9 percent in 2012 to 82.6 percent in 2016, likely reflecting the health insurance expansions associated with the Affordable Care Act. There were some declines in the unemployment rate (a 3.2 percentage point reduction from 2012 to 2016) and the percent of the county population in poverty (a 1.9 percentage point reduction from 2012 to 2016). These changes are indicative of the economic recovery during those years.

**Table 1:** Means for outcome variables and covariates.

	2008	2012	2016	2008–12 Difference	2012–16 Difference
*Outcome Variables*					
Democratic Vote Share [0–100]	52.95	51.11	48.92	-1.84***	-2.20***
Republican Vote Share [0–100]	45.72	47.29	46.13	1.57***	-1.16***
Other Vote Share [0–100]	1.38	1.60	4.95	0.22***	3.35***
Voter Turnout [0–100]	43.24	39.24	40.26	-3.99***	1.02***
*Covariates*					
Insurance Rate [0–100]	71.86	70.85	82.58	-1.01***	11.73***
County Unemployment Rate [0–100]	5.87	8.18	4.95	2.31***	-3.23***
Percent in Poverty [0–100]	13.25	15.97	14.09	2.72***	-1.89***
Percent of County Black [0–100]	13.52	13.79	14.05	0.27	0.25
Percent of County American Indian/Alaska Native [0–100]	1.27	1.36	1.39	0.09	0.03
Percent of County Asian/Pacific Islander [0–100]	5.32	5.81	6.39	0.50***	0.58***
Percent of County Hispanic [0–100]	15.74	16.92	17.88	1.17***	0.96**
County Median Household Income ($10k)	5.43	5.34	6.04	-0.09**	0.69***
Dem Campaign Expenditure Gap, 100k	12.36	13.38	1.43	1.02	-11.95**
Total Campaign Expenditures, 100k	46.74	41.86	47.55	-4.88	5.69
County Population Density, 1k per sq mi	1.57	1.64	1.69	0.07	0.05

Notes: Authors' calculations based on 2008, 2012, and 2016 Guardian and Townhall election data, Small Area Health Insurance Estimates, Small Area Income and Poverty Estimates, Bureau of Labor Statistics, and SEER population data. Means are weighted by county population.

Difference between the two years is statistically significant with * *p* < 0.10, ** *p* < 0.05, *** *p* < 0.01.

Table 2 provides means of the outcome and control variables separately for counties in Medicaid expansion vs. non-expansion states. In the 2016 election, the Democratic vote share averaged 52.6 percent in the states that expanded Medicaid, declining 2.5 percentage points from the 2012 election. The Democratic vote share was only 43.1 percent in the states that did not expand Medicaid and had declined by 1.5 percentage points from the 2012 election. Whereas Republican vote share did not change significantly between 2012 and 2016 for counties in expansion states, the Republican vote share did drop significantly in non-expansion counties from 54.1 percent in 2012 to 52.3 percent in 2016. Meanwhile, insurance rates rose by 13.3 percentage points in expansion counties and only 9.4 percentage points in non-expansion counties, likely reflecting the positive impact of the Medicaid expansion on insurance rates. All means in Tables 1 and 2 are population-weighted.

**Table 2:** Means for outcome variables and covariates, medicaid expansion vs. non-expansion counties.

	Expansion Counties					Non-Expansion Counties				
	2008	2012	2016	2008–12 Difference	2012–16 Difference	2008	2012	2016	2008–12 Difference	2012–16 Difference
*Outcome Variables*										
Democratic Vote Share [0–100]	56.82	55.14	52.60	-1.69***	-2.53***	46.58	44.60	43.08	-1.98***	-1.52***
Republican Vote Share [0–100]	41.55	43.08	42.24	1.52***	-0.83	52.58	54.11	52.28	1.52***	-1.83***
Other Vote Share [0–100]	1.58	1.79	5.15	0.21***	3.36***	1.06	1.29	4.64	0.23***	3.35***
Voter Turnout [0–100]	43.69	38.66	40.05	-5.03***	1.39***	42.48	40.18	40.60	-2.30***	0.41
*Covariates*										
Insurance Rate [0–100]	73.93	73.13	86.38	-0.81***	13.25***	68.45	67.17	76.59	-1.28***	9.42***
County Unemployment Rate [0–100]	6.08	8.54	5.15	2.46***	-3.48***	5.51	7.59	4.78	2.08***	-2.81***
Percent in Poverty [0–100]	12.71	15.33	13.63	2.63***	-1.70***	14.14	17.01	14.81	2.87***	-2.20***
Percent of County Black [0–100]	11.51	11.73	11.94	0.22	0.20	16.83	17.13	17.39	0.30	0.26
Percent of County American Indian/Alaska Native [0–100]	1.31	1.41	1.45	0.10	0.03	1.21	1.28	1.29	0.07	0.01
Percent of County Asian/Pacific Islander [0–100]	6.78	7.37	8.07	0.60*	0. 69**	2.91	3.29	3.73	0.38***	0.44**
Percent of County Hispanic [0–100]	15.99	17.12	18.01	1.13**	0.89	15.33	16.57	17.67	1.25**	1.09*
County Median Household Income ($10k)	5.69	5.60	6.35	-0.09*	0.75***	5.00	4.93	5.54	-0.07	0.61***
Dem Campaign Expenditure Gap, 100k	20.00	23.25	14.62	3.26	-8.63	-0.22	-2.58	-19.46	-2.37*	-16.87***
Total Campaign Expenditures, 100k	63.14	60.11	50.19	-3.03	-9.92	19.71	12.35	43.37	-7.36***	31.02***
County Population Density, 1k per sq mi	2.09	2.19	2.25	0.09	0.07	0.69	0.75	0.80	0.05*	0.05

Notes: Authors' calculations based on 2008, 2012, and 2016 Guardian and Townhall election data, Small Area Health Insurance Estimates, Small Area Income and Poverty Estimates, Bureau of Labor Statistics, and SEER population data. Means are weighted by county population.

Difference between the two years is statistically significant with * *p* < 0.10, ** *p* < 0.05, *** *p* < 0.01.

**Fig 2:**
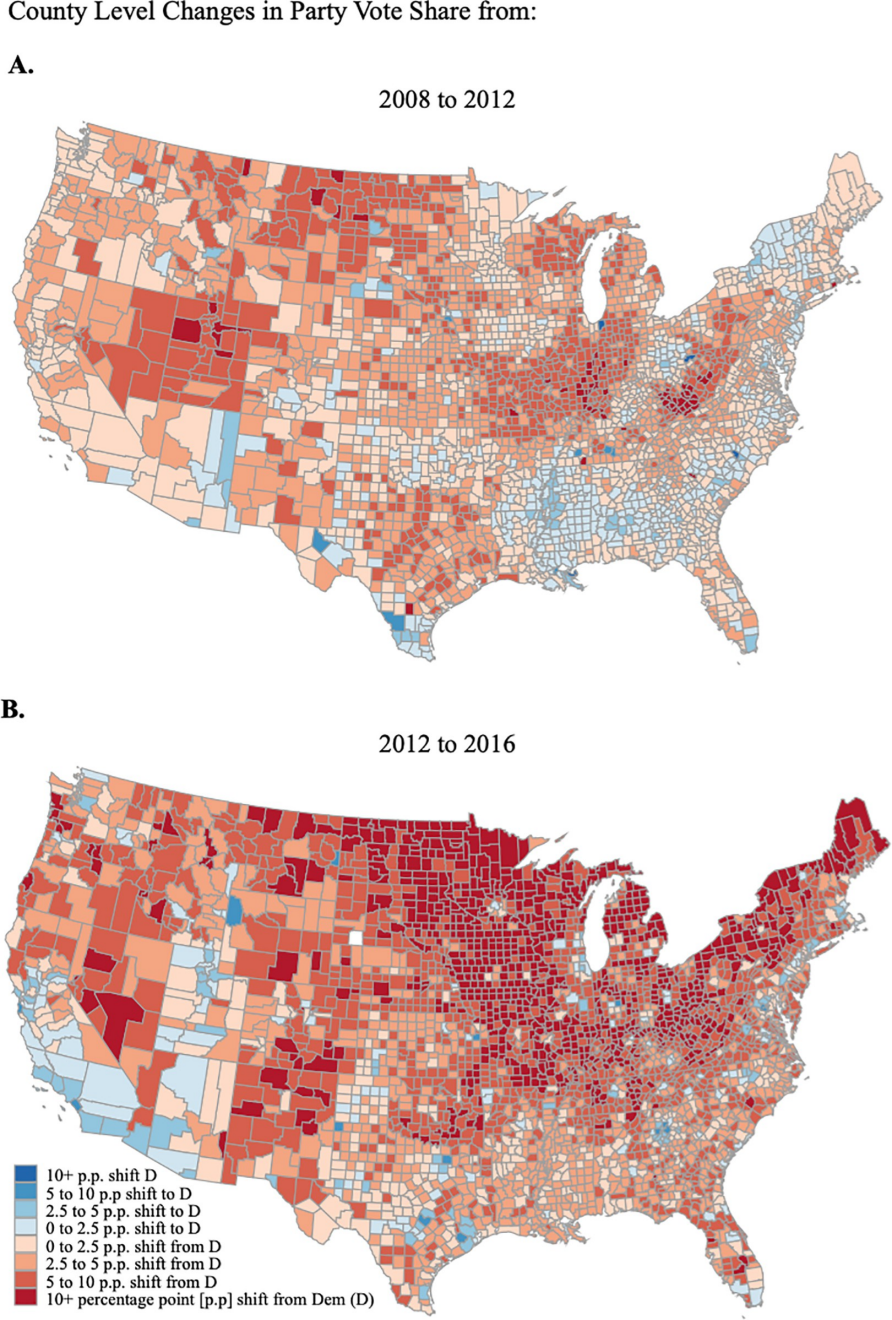
County-level changes in party vote share in 2012 and 2016.

A pair of maps illustrates the changes in Democratic vote share between the 2008 and 2012 presidential elections and the 2012 and 2016 presidential elections (Fig 2). In the most recent election, the Democrats experienced the greatest gains in the Pacific Northwest, California, Utah, and urban areas of the South, but lost the most vote share in the Midwestern states, Pennsylvania, and upstate New York.

**Fig 3:**
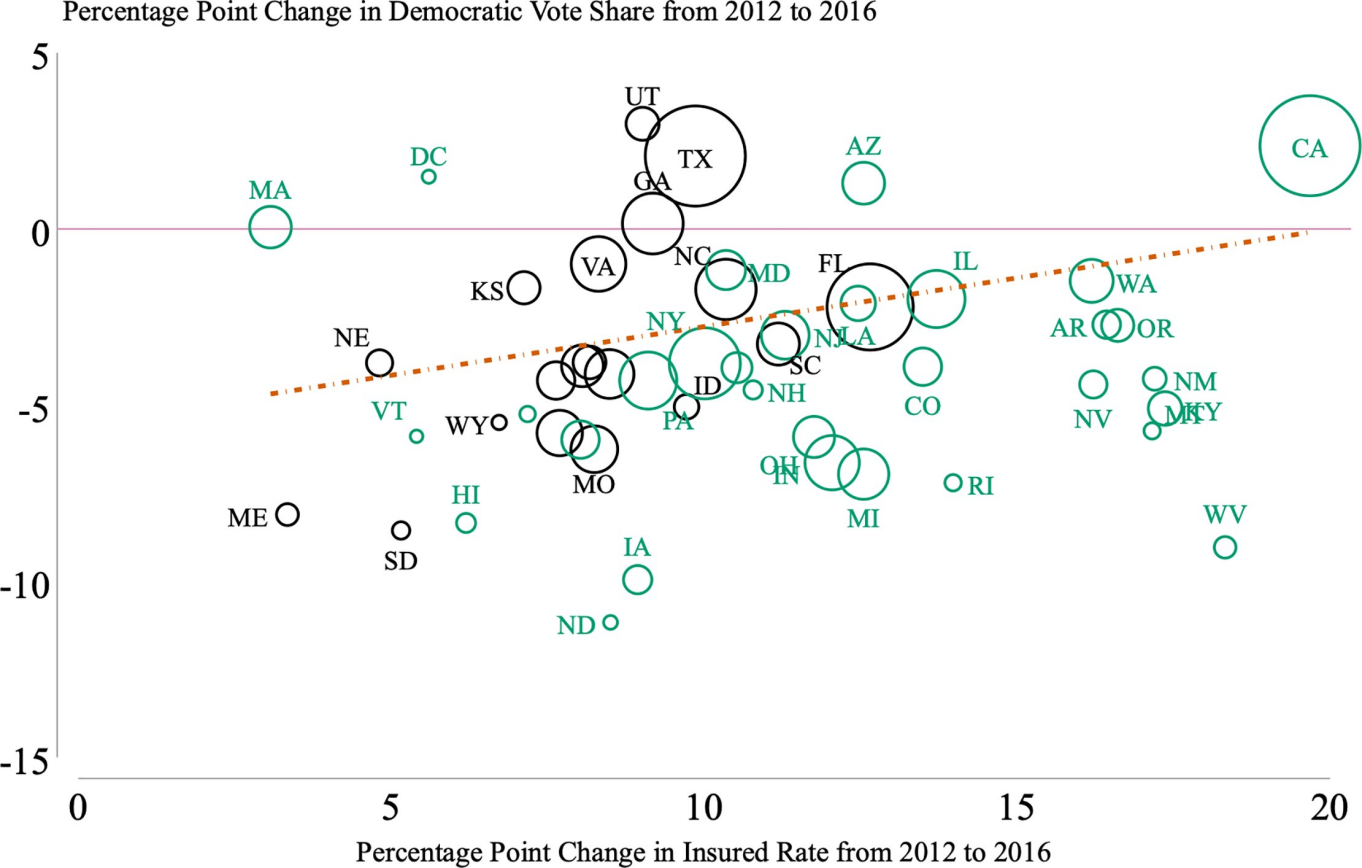
Increases in insurance are associated with increases in democratic vote share. The size of each circle is proportional to state population. The dashed line represents a best-fit line weighted by population. The slope of this best-fit line is 0.32, meaning that at the state-level a one percentage point increase in the insurance rate from 2012 to 2016 was associated with a 0.32 percentage point increase (p<0.01, 95% CI [0.09, 0.55]) in the Democratic vote from 2012 to 2016. Note that this is an unconditional correlation at the state-level, our county-level analysis controls for additional confounders, county fixed-effects, and year fixed-effects. States that expanded Medicaid are displayed in green. Medicaid expansion status is as reported by the Kaiser Family Foundation in Nov 2016.

Fig 3 illustrates the unconditional state-level relationship between the percentage point change in Democratic vote share from 2012 to 2016 and percentage point change in health insurance coverage over the same period. The dashed line displays the best-fit population weighted line and indicates that there is a positive relationship between increases in insurance at the state-level and increases in Democratic vote share at the state-level. The slope of this best-fit line is 0.32, meaning that at the state-level, a one percentage point increase in the insurance rate from 2012 to 2016 was associated with a 0.32 percentage point increase (p<0.01, 95% CI [0.09, 0.55]) in the Democratic vote from 2012 to 2016. Note that this is an unconditional correlation at the state-level, our county-level analysis controls for additional confounders.

## Regression results

We next present least squares regression results from data at the county-level that control for county fixed-effects, year fixed-effects, and a variety of observable and time-varying county characteristics. Table 3 presents regression results for all U.S. counties. It indicates that a one percentage point increase in county health insurance coverage was associated with a 0.25 percentage point increase in the vote share of the Democratic presidential candidate, which is statistically significant at the 5% level. Subsequent columns of Table 3 indicate that these gains on the part of the Democratic candidate came almost fully at the expense of the Republican (as opposed to third-party) presidential candidates. The final column of Table 3 indicates that voter turnout was not significantly associated with changes in the county health insurance rate.

**Table 3:** Regression results showing association between county insurance rate and voting patterns.

	Democratic Vote Share	GOP Vote Share	Other Party Vote Share	Voter Turnout
Insurance Rate [0–100]	0.25** (0.10)	-0.24** (0.10)	0.00 (0.03)	-0.04 (0.04)
County Unemployment Rate [0–100]	0.43* (0.23)	-0.54** (0.24)	0.16 (0.10)	-0.23 (0.14)
Percent in Poverty [0–100]	-0.02 (0.10)	0.08 (0.09)	-0.02 (0.04)	0.11 (0.09)
Percent of County Black [0–100]	0.52 (0.33)	-0.53 (0.32)	0.04 (0.07)	0.04 (0.23)
Percent of County American Indian/Alaska Native [0–100]	-4.38*** (1.51)	2.58* (1.37)	1.60*** (0.55)	-1.17 (0.96)
Percent of County Asian/Pacific Islander [0–100]	2.31*** (0.33)	-2.27*** (0.36)	-0.02 (0.16)	-0.96*** (0.30)
Percent of County Hispanic [0–100]	1.09*** (0.21)	-0.98*** (0.20)	-0.08 (0.08)	-0.04 (0.20)
County Median Household Income ($10k)	0.98* (0.50)	-1.88*** (0.65)	0.93* (0.53)	0.88 (0.61)
Dem Campaign Expenditure Gap, 100k	-0.00 (0.00)	0.00 (0.00)	0.00 (0.00)	-0.00 (0.00)
Total Campaign Expenditures, 100k	0.00 (0.00)	-0.00 (0.00)	-0.00 (0.00)	0.00* (0.00)
Population Density, 1k per sq. mi	4.49*** (1.59)	-3.40** (1.67)	-1.01*** (0.19)	-0.82 (0.64)
Observations	9,327	9,327	9,327	9,327
Adjusted R-squared	0.963	0.930	0.624	0.878

Notes: Authors' calculations based on 2008, 2012, and 2016 Guardian and Townhall election data, Small Area Health Insurance Estimates, Small Area Income and Poverty Estimates, Bureau of Labor Statistics, and SEER population data. Observations are weighted by county population. The covariate Insurance Rate refers to the percent of the county’s working-age population (ages 18–64) below 400% FPL with health insurance. The regression is a weighted-least squares specification that also includes year fixed-effects and county fixed-effects. Standard errors are clustered at the state-level, and the regression is weighted by county population.

* *p* < 0.10

** *p* < 0.05

*** *p* < 0.01

We also estimate models separately for states that did and did not expand Medicaid; these results can be found in Table 4. In both types of states, gains in health insurance coverage were associated with increases in the vote share of the Democratic presidential candidate. Specifically, a one-percentage-point increase in coverage was associated with an increase in Democratic vote share of 0.36 percentage points in the expansion states and 0.36 percentage points in the non-expansion states. Using a Chow test, we find that the difference between these coefficients across the two samples is not statistically significant.

**Table 4:** Regression results for medicaid expansion vs. non-expansion counties.

	Expansion Counties				Non-Expansion Counties			
	Democratic Vote Share	GOP Vote Share	Other Party Vote Share	Voter Turnout	Democratic Vote Share	GOP Vote Share	Other Party Vote Share	Voter Turnout
Insurance Rate [0–100]	0.36*** (0.12)	-0.37*** (0.12)	0.02 (0.03)	-0.10*^, c^ (0.05)	0.36*** (0.05)	-0.29*** (0.06)	-0.03 (0.04)	0.21*** (0.05)
County Unemployment Rate [0–100]	-0.00^c^ (0.24)	-0.06 ^c^ (0.24)	0.07 (0.09)	-0.38** (0.17)	1.00*** (0.22)	-1.18*** (0.26)	0.27 (0.21)	0.22 (0.14)
Percent in Poverty [0–100]	0.26***^, c^ (0.08)	-0.15**^, a^ (0.07)	-0.08***^, b^ (0.03)	0.12 (0.12)	-0.12* (0.06)	0.04 (0.08)	0.12 (0.07)	-0.04 (0.05)
Percent of County Black [0–100]	0.12 (0.27)	-0.30 (0.28)	0.19*^, b^ (0.09)	0.14 (0.37)	0.71* (0.38)	-0.57 (0.36)	-0.11 (0.07)	-0.16 (0.11)
Percent of County American Indian/Alaska Native [0–100]	-2.81 (1.94)	0.88 ^a^ (1.79)	1.85** (0.77)	-0.82 (1.00)	-5.69*** (1.64)	4.49*** (1.22)	1.07* (0.55)	0.05 (0.77)
Percent of County Asian/Pacific Islander [0–100]	2.35*** (0.42)	-2.44*** (0.45)	0.02 (0.13)	-0.82*** (0.29)	1.97*** (0.62)	-1.72** (0.61)	0.00 (0.34)	-0.71** (0.30)
Percent of County Hispanic [0–100]	1.36***^, c^ (0.24)	-1.26***^, c^ (0.25)	-0.12 (0.09)	-0.21 (0.14)	0.30 (0.22)	-0.09 (0.24)	-0.11 (0.16)	0.12 (0.21)
County Median Household Income ($10k)	2.15***^, b^ (0.64)	-2.28*** (0.66)	0.24 (0.23)	0.99 ^a^ (0.61)	0.42 (0.49)	-3.20* (1.80)	2.57 (1.80)	-0.45 (0.45)
Dem Campaign Expenditure Gap, 100k	0.00 (0.00)	-0.00 (0.00)	0.00**^, a^ (0.00)	-0.00***^, c^ (0.00)	-0.00 (0.00)	0.00 (0.00)	-0.00 (0.00)	0.00*** (0.00)
Total Campaign Expenditures, 100k	-0.00***^, b^ (0.00)	0.00***^, b^ (0.00)	-0.00 (0.00)	0.00*^, c^ (0.00)	0.00 (0.00)	-0.00 (0.00)	-0.00 (0.00)	0.00*** (0.00)
Population Density, 1k per sq. mi	4.00***^, b^ (0.81)	-3.06***^, b^ (0.81)	-0.95***^, c^ (0.12)	-0.56 ^b^ (0.70)	12.96*** (3.96)	-13.99*** (4.44)	1.03* (0.50)	-2.93*** (0.90)
Observations	4,404	4,404	4,404	4,404	4,923	4,923	4,923	4,923
Adjusted R-squared	0.969	0.966	0.840	0.867	0.956	0.874	0.487	0.920

Notes: Authors' calculations based on 2008, 2012, and 2016 Guardian and Townhall election data, Small Area Health Insurance Estimates, Small Area Income and Poverty Estimates, Bureau of Labor Statistics, and SEER population data. Means are weighted by county population. The covariate Insurance Rate refers to the percent of the county’s working-age population (ages 18–64) below 400% FPL with health insurance. The regression is a weighted-least squares specification that also includes year fixed-effects and county fixed-effects. Standard errors are clustered at the state-level, and the regression is weighted by county population.

* *p* < 0.10

** *p* < 0.05

*** *p* < 0.01

Coefficient for expansion county regressions is significantly different from that of non-expansion county regressions with ^a^ *p* < 0.10, ^b^ *p* < 0.05, ^c^ *p* < 0.01.

A notable difference between expansion and non-expansion states is that increases in health insurance coverage are not significantly associated with increased voter turnout in expansion states, but are associated with significantly higher turnout in non-expansion states. Others who study the effects of health insurance on voter turnout find mixed results. Baicker & Finkelstein (2018) used data from the Oregon Health Insurance Experiment and found that Medicaid expansion increased voter turnout in the 2008 presidential election [15]. Haselswerdt (2017) used voter data for all 435 US House races in 2014 and 2012 and found that Medicaid expansion was associated with increased voter turnout [10]. Our findings contrast with Haselswerdt’s earlier work, suggesting that voters may respond differently during presidential elections relative to other elections, or during different election cycles. Indeed, Clinton and Sances (2018) study the effect of the ACA Medicaid expansion on voter turnout in both 2014 and 2016 and find that the expansion increased voter turnout in the 2014 election but had no significant impact in the 2016 election [9].

Even though the difference between expansion and non-expansion counties is not statistically significant, we explore a potential reason for this difference. The regression results presented in Table 3 assume that the relationship between increases in insurance and Democratic vote share is linear across the entire distribution of potential insurance increases. As evidenced in Fig 1, those states that expanded Medicaid experienced a significant increase in the percentage of those with health insurance. If the relationship between insurance coverage and Democratic vote share exhibits a diminishing effect, then it is possible that the difference in coefficients is due to this diminishing relationship. Table 5 presents results that are consistent with this theory.

**Table 5:** Medicaid expansion effect is same as the high insurance effect, implying diminishing returns.

	Baseline Model	Baseline + Medicaid Expansion Indicator	Baseline + High Insurance Indicator	Baseline + High Insurance Indicator AND Drop Medicaid Expansion States
Insurance Rate [0–100]	0.25** (0.10)	0.39*** (0.10)	0.34*** (0.08)	0.35*** (0.05)
County Unemployment Rate [0–100]	0.43* (0.23)	0.37* (0.21)	0.43** (0.19)	1.01*** (0.22)
Percent in Poverty [0–100]	-0.02 (0.10)	0.13* (0.07)	0.09 (0.07)	-0.09 (0.06)
Percent of County Black [0–100]	0.52 (0.33)	0.50* (0.30)	0.60** (0.29)	0.72* (0.36)
Percent of County American Indian/Alaska Native [0–100]	-4.38*** (1.51)	-3.97*** (1.31)	-4.35*** (1.22)	-5.75*** (1.50)
Percent of County Asian/Pacific Islander [0–100]	2.31*** (0.33)	2.46*** (0.34)	2.28*** (0.31)	1.89*** (0.61)
Percent of County Hispanic [0–100]	1.09*** (0.21)	0.99*** (0.21)	0.80*** (0.19)	0.23 (0.21)
County Median Household Income ($10k)	0.98* (0.50)	1.55*** (0.48)	1.61*** (0.44)	0.67 (0.50)
Dem Campaign Expenditure Gap, 100k	-0.00 (0.00)	-0.00 (0.00)	-0.00 (0.00)	-0.00 (0.00)
Total Campaign Expenditures, 100k	0.00 (0.00)	-0.00 (0.00)	0.00 (0.00)	0.00 (0.00)
Population Density, 1k per sq. mi	4.49*** (1.59)	4.65*** (1.31)	4.43*** (1.03)	12.32*** (3.81)
Expansion X Post-2014		-3.28*** (0.81)		
Indicator for Highest Ins. Quintile			-3.51*** (0.52)	-2.04** (0.72)
Observations	9,327	9,327	9,327	4,923
Adjusted R-squared	0.963	0.965	0.967	0.956

Note: Authors' calculations based on 2008, 2012, and 2016 Guardian and Townhall election data, Small Area Health Insurance Estimates, Small Area Income and Poverty Estimates, Bureau of Labor Statistics, and SEER population data. The covariate Insurance Rate refers to the percent of the county’s working-age population (ages 18–64) below 400% FPL with health insurance. The regression is a weighted-least squares specification that also includes year fixed-effects and county fixed-effects. Columns 3 and 4 include state fixed-effects instead of county fixed-effects. Standard errors are clustered at the state-level, and the regression is weighted by county population

* *p* < 0.10

** *p* < 0.05

*** *p* < 0.01

The first column of Table 5 is our baseline result. The second column presents results from a specification that adds a Medicaid expansion indicator to our baseline model. These results initially seem to suggest that Medicaid expansion dampens the effect of insurance increases on changes to Democratic vote share. (Note it does not imply a negative effect despite the negative coefficient since expansions are associated with larger increases in insurance coverage. Therefore, the total effect is still positive, but smaller that if it would have been from an insurance increase not due to Medicaid expansion.) However since Medicaid expansion states have higher levels of insurance than non-expansion states (see Fig 1) due to the expansion, it is possible that this is not an expansion effect, but evidence of a diminishing return to insurance increases. To explore this possibility, the third column of Table 5 includes an indicator if a county’s percent with insurance is in the top twenty percentiles. The coefficient on this indicator is not statistically different than that on the Medicaid expansion binary variable. A reason for this similarity could be that the indicator is highly correlated between the two. That is, perhaps Medicaid expansion states are also the same states with high rates of insurance. To rule out the possibility that Medicaid expansion is driving the findings, we run the same specification dropping all Medicaid expansion states from the analysis. The coefficient is remarkably stable, indicating that there is likely a diminishing return to insurance increases on Democratic vote share.

As a robustness check to ensure that these relationships would be found no matter which earlier presidential election cycle we compared with 2016, we drop 2012 from the sample (i.e. we compare 2016 to 2008) and alternately drop 2008 from the sample (i.e. we compare 2016 to 2012). The results, in Table 6, indicate that the main result is robust: counties that experienced greater gains in health insurance coverage also saw larger increases in the vote share for the Democratic candidate, and this increase came almost entirely at the expense of the Republican candidate as opposed to third-party candidates.

**Table 6:** Sensitivity analyses dropping one year at a time from analysis.

	Omit Year 2008				Omit Year 2012			
	Democratic Vote Share	GOP Vote Share	Other Party Vote Share	Voter Turnout	Democratic Vote Share	GOP Vote Share	Other Party Vote Share	Voter Turnout
Insurance Rate [0–100]	0.25** (0.10)	-0.25** (0.11)	0.01 (0.04)	0.07* (0.04)	0.23** (0.11)	-0.22** (0.11)	0.02 (0.03)	-0.17* (0.09)
County Unemployment Rate [0–100]	0.66** (0.26)	-0.95*** (0.27)	0.29* (0.16)	-0.23** (0.10)	0.38 (0.35)	-0.36 (0.35)	0.06 (0.12)	0.17 (0.17)
Percent in Poverty [0–100]	-0.44*** (0.11)	0.53*** (0.12)	-0.09 (0.09)	0.34*** (0.10)	-0.04 (0.18)	0.23 (0.16)	-0.02 (0.06)	-0.18 (0.16)
Percent of County Black [0–100]	0.83** (0.40)	-0.82** (0.35)	-0.01 (0.19)	-0.75*** (0.16)	0.44 (0.33)	-0.49 (0.33)	0.08 (0.07)	-0.11 (0.10)
Percent of County American Indian/Alaska Native [0–100]	-6.66*** (1.92)	4.29** (1.88)	2.37*** (0.70)	0.51 (0.82)	-4.10*** (1.49)	2.09 (1.38)	1.76*** (0.57)	-0.82 (0.75)
Percent of County Asian/Pacific Islander [0–100]	3.09*** (0.67)	-2.77*** (0.81)	-0.32 (0.35)	0.16 (0.29)	2.61*** (0.35)	-2.58*** (0.40)	-0.07 (0.19)	-0.52** (0.24)
Percent of County Hispanic [0–100]	1.50*** (0.39)	-1.35*** (0.40)	-0.15 (0.19)	0.48*** (0.18)	1.17*** (0.23)	-1.07*** (0.22)	-0.07 (0.08)	0.07 (0.20)
County Median Household Income ($10k)	1.87** (0.85)	-3.27** (1.32)	1.40* (0.82)	0.92** (0.37)	-0.60 (0.84)	-0.30 (0.93)	1.23* (0.73)	-1.10* (0.56)
Dem Campaign Expenditure Gap, 100k	-0.00 (0.00)	0.00 (0.00)	0.00* (0.00)	0.00 (0.00)	-0.00 (0.00)	0.00 (0.00)	-0.00 (0.00)	-0.00 (0.00)
Total Campaign Expenditures, 100k	-0.00 (0.00)	0.00 (0.00)	0.00 (0.00)	0.00* (0.00)	0.00 (0.00)	-0.00 (0.00)	0.00 (0.00)	0.00 (0.00)
Population Density, 1k per sq. mi	6.55** (2.62)	-4.24 (2.64)	-2.32*** (0.44)	1.04 (1.20)	4.65*** (1.69)	-3.52* (1.78)	-1.03*** (0.19)	-0.74 (0.48)
Observations	6,218	6,218	6,218	6,218	6,218	6,218	6,218	6,218
Adjusted R-squared	0.973	0.958	0.573	0.958	0.948	0.904	0.594	0.842

Notes: Authors' calculations based on 2008, 2012, and 2016 Guardian and Townhall election data, Small Area Health Insurance Estimates, Small Area Income and Poverty Estimates, Bureau of Labor Statistics, and SEER population data. Means are weighted by county population. The covariate Insurance Rate refers to the percent of the county’s working-age population (ages 18–64) below 400% FPL with health insurance. The regression is a weighted least squares specification that includes year fixed-effects and county fixed-effects. Standard errors are clustered at the state-level, and the regression is weighted by county population.

* *p* < 0.10

** *p* < 0.05

*** *p* < 0.01

S1 Table presents results from a regression model that provides suggestive evidence on the relationship between insurance premiums and election results. We find that the Democratic presidential candidate in 2016 underperformed that in 2012 in areas that experienced higher-than average increases in health insurance premiums. A one percent increase in a county’s marketplace premiums was associated with a 0.02 percentage point decrease in the change in the county’s Democratic vote share. In other words, Clinton tended to underperform Obama in counties where marketplace premiums rose more than average. In contrast, the relationship between premium changes and GOP vote share changes was positive, though not statistically significant.

S1 Fig explores a check of our identification assumption that those counties that experienced gains in insurance would have continued to vote a similar share Democratic in the absence of changes in insurance status. S1 Fig indicates that while counties who saw the largest increases in insurance over our sample did tend to vote more Democratic on average, they were not doing so in a manner that overtly violates the parallel trends assumption needed for identification. However, it should be noted that a finding of parallel trends in the pre-policy treatment is not necessarily evidence that parallel trends would have continued in the absence of the policy.

## Discussion

This paper provides the first systematic analysis of how insurance gains at the county-level are associated with Democratic vote share changes. We find that gains in health insurance coverage were associated with greater increases in the share of votes for the Democratic presidential candidate. Thus, the Democratic candidate fared better in counties where the ACA had greater positive impact; this is true both in states that did and did not expand Medicaid. Our findings are consistent with prior work that demonstrates that attachment to U.S. political parties is a strong predictor of attitudes towards health care change [16].

The ACA led to substantial increases in health insurance coverage nationwide [17], which, if the correlations we report reflect a causal relationship, helped the Democratic candidate. Clearly, many other factors influenced the presidential election, but these results clarify how changes in health insurance coverage correlate with voter behavior and election results.

This study, like all such studies, has limitations that warrant consideration. We reiterate that elections are the results of millions of individual decisions, each of which is based on numerous factors, and while we are interested in exploring the association of voting outcomes with health insurance changes, we are wary of overly simplistic explanations for highly complex events. Our methodology can only test for associations; it cannot prove causation. We cannot know whether changes in health insurance coverage rates caused people to change their vote as it is possible that factors not controlled for in the model are correlated with both coverage and vote results, causing omitted variable bias in our estimates. However, we control for a number of economic and demographic factors and find these associations to be robust.

Republican proposals to repeal and replace the ACA would reduce health insurance coverage [18]. Alternatively, the program could be managed in such a way as to reduce health insurer participation and thus rates of health insurance coverage. Should these events come about, an important direction for further research would be to examine whether future voting patterns at the county-level correlate with the magnitude of the increase in uninsurance rates.

## Supporting information

S1 File Relationship between insurance premiums and election results. (DOCX)

S2 File Relationship between gains in insurance in 2016 and vote share in 2008 and 2012. (DOCX)

S1 Table Regression results showing association between change in county insurance premiums and change in voting patterns from 2012 to 2016. Authors' calculations based on 2012 and 2016 Guardian and Townhall election data, Small Area Health Insurance Estimates, Small Area Income and Poverty Estimates, Bureau of Labor Statistics, and SEER population data. Observations are weighted by county population. The covariate Insurance Rate refers to the percent of the county’s working-age population (ages 18–64) below 400% FPL with health insurance. The regressions are weighted-least squares specifications that also includes year fixed effects and state fixed effects. Standard errors are robust, and the regression is weighted by county population. * *p* < 0.10, ** *p* < 0.05, *** *p* < 0.01.
(DOCX)

S1 Fig Difference in democratic vote share between counties by change in insurance. (TIF)
