# Supplementary material for: Gains in health insurance coverage explain variation in Democratic vote share in the 2008-2016 presidential elections
Source: PLoS One. 2019 Apr 4;14(4):e0214206. doi: 10.1371/journal.pone.0214206 (PMC6449023; doi:10.1371/journal.pone.0214206)
Supplement: S1 File — (DOCX) [file pone.0214206.s001.docx]

**S1. Relationship between Insurance Premiums and Election Results**

In this section, we provide suggestive evidence on the relationship between insurance premiums and election results by estimating models using county-level data, in which the change between 2012 and 2016 in the Republican share of the presidential vote is regressed on the recent change in ACA marketplace premiums and the change in health insurance coverage, controlling for a host of possible confounders at the state and county-levels. To clarify, the outcome we examine is whether the Republican presidential candidate in 2016 received a higher share of the vote than the Republican presidential candidate in 2012; that is, did Trump outperform Romney in a given county, as a function of changes in health insurance markets in that county.  We also estimate models of whether the Democratic presidential candidate performed better in 2016 than in 2012, and also models of other presidential candidates and voter turnout.

The key independent variables in those regressions are 1) the change in the insurance rate in the county after the ACA (measured as the change from 2013 to 2014 in insurance rate for people aged 18-64 years below 400% of the federal poverty level (FPL)), and 2) the change in the county’s ACA marketplace premium between 2014 and 2017 (measured as the percent change in premium for the second-lowest silver plan available in the county).  We collected data on county ACA marketplace premiums from Healthcare.gov, which covers the states that use the federal marketplace architecture. We have ACA premiums data for counties in the 34 states that participate in the federal marketplaces since 2014, but not the 16 states and DC that run their own Marketplace. Our model further controls for a variety of other possible confounders at the county-level, including the county’s unemployment rate, median income, percent of population in poverty, percent of population that is non-White, population density, campaign spending, state fixed effects, and year fixed effects. All specifications include robust standard-errors.

The regression results are displayed in S1 Table. We find that the Democratic presidential candidate in 2016 underperformed that in 2012 in areas that experienced higher-than average increases in health insurance premiums. A one percent increase in a county’s marketplace premiums was associated with a (statistically insignificant) 0.02 percentage point decrease in the change in the county’s Democratic vote share. In other words, Clinton tended to underperform Obama in counties in which marketplace premiums rose more than average. In contrast, the relationship between premium changes and GOP vote share changes was positive, though not statistically significant. For other presidential candidates, a one percent increase in a county’s marketplace premiums was associated with a 0.01 percentage point increase in the change in the county’s vote share. There was no significant relationship between premium changes and changes in voter turnout.
