# Supplementary material for: Gains in health insurance coverage explain variation in Democratic vote share in the 2008-2016 presidential elections
Source: PLoS One. 2019 Apr 4;14(4):e0214206. doi: 10.1371/journal.pone.0214206 (PMC6449023; doi:10.1371/journal.pone.0214206)
Supplement: S2 File — (DOCX) [file pone.0214206.s002.docx]

**S2. Relationship between Gains in Insurance in 2016 and Vote Share in 2008 and 2012**

In this section we explore a check of our identification assumption that those counties who experienced gains in insurance would have continued to vote a similar share Democratic in the absence of the insurance expansion. These checks are displayed in S1 Fig A and B. In this section, we consider the differences in vote share for presidential elections in 2008 and 2012 to be in the “pre-period” and the counties who gained the most insurance from 2012 to 2016 to be the “treated” group. In S1 Fig A, we compare the difference in Democratic vote share in 2008, 2012, and 2016 from counties that gained the most (the top 20%) in insurance coverage from 2012 to 2016 to those counties that gained the least (the bottom 20%) from 2012 to 2016. In S1 Fig B, we report the comparison between the top 20% and the bottom 80% to show that the same pattern holds when the whole sample is considered.

We choose those counties that had the largest gain from 2012 to 2016 to construct our comparison group since this will roughly approximate a “treated” group in a typical difference-in-differences analysis. We choose 2016 as the “post-period” and 2008 and 2012 as the “pre-period” since there were substantial insurance gains from 2014 to 2016 in many counties due to the Affordable Care Act. By examining the trend in the difference between our “treated” and “control” groups in the “pre-period,” we are able to show that those who received the largest insurance increases in the “post-period” did not overtly appear to be differentially trending to be more Democratic across time. That is, we are able to show that the findings reflected in our coefficient estimates are likely not due to differential trends between the most treated and the least treated counties that would not be accounted for by county fixed effects. Any time-invariant pre-period differences in county-level vote between the “treated” and “control” counties will be absorbed by county fixed-effects in our specification of interest. We note, however, that a finding of parallel trends in the pre-policy treatment is not necessarily evidence that parallel trends would have continued in the absence of the policy.

S1 Fig A and B show that while those counties who saw the largest increases in insurance coverage from 2012 to 2016 tended to be more Democratic than Republican in the “pre-period” (an average difference in Democratic vote share of around 6%), there are not concerning differential pre-period trends in 2008 and 2012. This figure also shows that following the expansion of insurance in 2014, the difference in Democratic vote share increases to 8 percent, an increase of 2 over percentage points from the average 2012 difference. This second point is consistent with our overall finding. This figure demonstrates that there are not overt, worrisome non-parallel trends in our research setting.
