# Supplementary material for: Gains in health insurance coverage explain variation in Democratic vote share in the 2008-2016 presidential elections
Source: PLoS One. 2019 Apr 4;14(4):e0214206. doi: 10.1371/journal.pone.0214206 (PMC6449023; doi:10.1371/journal.pone.0214206)
Supplement: S1 Table — Authors' calculations based on 2012 and 2016 Guardian and Townhall election data, Small Area Health Insurance Estimates, Small Area Income and Poverty Estimates, Bureau of Labor Statistics, and SEER population data. Observations are weighted by county population. The covariate Insurance Rate refers to the percent of the county’s working-age population (ages 18–64) below 400% FPL with health insurance. The regressions are weighted-least squares specifications that also includes year fixed effects and state fixed effects. Standard errors are robust, and the regression is weighted by county population. * p < 0.10, ** p < 0.05, *** p < 0.01. (DOCX) [file pone.0214206.s003.docx]

**S1 Table. Regression Results Showing Association between Change in County Insurance Premiums and Change in Voting Patterns from 2012 to 2016**

|  | Democrat Vote Share | GOP Vote Share | Other Party Vote Share | Voter Turnout |
| --- | --- | --- | --- | --- |
| Percent Change in ACA Premiums [0-100], 2014 to 2016 | -0.021 (0.014) | 0.012 (0.015) | 0.009^***^ (0.003) | -0.013 (0.015) |
|  |  |  |  |  |
| Change in Insurance Rate [0-100], 2013 to 2014 | 0.078 (0.048) | -0.167^***^ (0.058) | 0.089^***^ (0.022) | 0.035 (0.041) |
|  |  |  |  |  |
| Change in Unemployment Rate [0-100], 2012 to 2016 | 0.622^***^ (0.107) | -0.684^***^ (0.126) | 0.062^*^ (0.036) | -0.223^***^ (0.076) |
|  |  |  |  |  |
| Unemployment Rate [0-100] | -0.638^***^ (0.181) | 0.798^***^ (0.217) | -0.160^***^ (0.043) | 0.298^***^ (0.070) |
|  |  |  |  |  |
| Percent in Poverty [0-100] | 0.077 (0.051) | -0.046 (0.058) | -0.031^**^ (0.014) | 0.088^**^ (0.036) |
|  |  |  |  |  |
| Percent of County Black [0-100] | 0.131^***^ (0.013) | -0.131^***^ (0.014) | 0.000 (0.003) | -0.092^***^ (0.009) |
|  |  |  |  |  |
| Percent of County American Indian/Alaska Native [0-100] | 0.003 (0.028) | -0.021 (0.034) | 0.018 (0.011) | -0.051^***^ (0.018) |
|  |  |  |  |  |
| Percent of County Asian/Pacific Islander [0-100] | -0.000 (0.043) | 0.044 (0.048) | -0.044^***^ (0.010) | 0.004 (0.035) |
|  |  |  |  |  |
| Percent of County Hispanic [0-100] | 0.099^***^ (0.011) | -0.108^***^ (0.013) | 0.009^***^ (0.003) | -0.006 (0.010) |
|  |  |  |  |  |
| County Median Household Income ($10k) | 1.652^***^ (0.195) | -1.826^***^ (0.223) | 0.174^***^ (0.066) | 0.475^**^ (0.195) |
|  |  |  |  |  |
| Dem Campaign Expenditure Gap, 100k | -0.001 (0.001) | 0.001 (0.001) | -0.000 (0.000) | 0.003^**^ (0.001) |
|  |  |  |  |  |
| Total Campaign Expenditures, 100k | -0.000 (0.001) | 0.000 (0.001) | -0.000 (0.000) | 0.003^**^ (0.001) |
|  |  |  |  |  |
| County Population Density, 1k per sq mi | 0.115^***^ (0.033) | -0.094^**^ (0.039) | -0.021^*^ (0.012) | -0.021 (0.036) |
| Observations | 3,108 | 3,108 | 3,108 | 3,108 |
| Adjusted R-squared | 0.746 | 0.756 | 0.848 | 0.482 |

Notes: Authors' calculations based on 2012 and 2016 Guardian and Townhall election data, Small Area Health Insurance Estimates, Small Area Income and Poverty Estimates, Bureau of Labor Statistics, and SEER population data. Observations are weighted by county population. The covariate Insurance Rate refers to the percent of theee county’s working-age population (ages 18-64) below 400% FPL with health insurance. The regressions are weighted-least squares specifications that also includes year fixed effects and state fixed effects. Standard errors are robust, and the regression is weighted by county population.

^*^ *p* < 0.10, ^**^ *p* < 0.05, ^***^ *p* < 0.01
