# Supplementary figures and images for: Gains in health insurance coverage explain variation in Democratic vote share in the 2008-2016 presidential elections
Source: PLoS One. 2019 Apr 4;14(4):e0214206. doi: 10.1371/journal.pone.0214206 (PMC6449023; doi:10.1371/journal.pone.0214206)

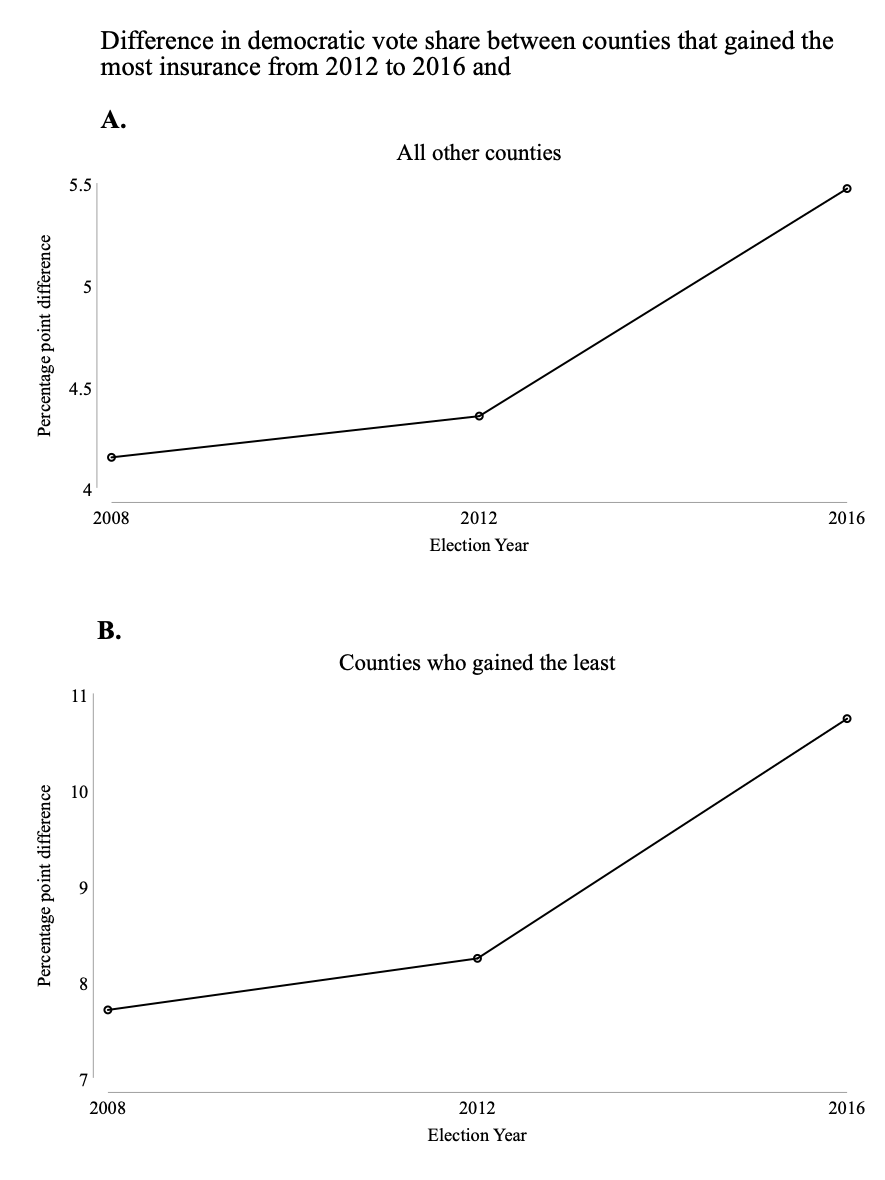

Supplement: S1 Fig — (TIF) [file pone.0214206.s004.tif]
